# Purification and characterization of a hyaluronidase from venom of the spider *Vitalius dubius* (Araneae, Theraphosidae)

**DOI:** 10.1186/1678-9199-20-2

**Published:** 2014-02-04

**Authors:** Rafael Sutti, Mariana Leite Tamascia, Stephen Hyslop, Thomaz Augusto Alves Rocha-e-Silva

**Affiliations:** 1 Department of Physiological Sciences, Santa Casa de São Paulo Medical School, Rua Cesário Motta Jr., 61, Vila Buarque, CEP 01.221-020 São Paulo, SP, Brasil; 2 Department of Pharmacology, School of Medical Sciences, Federal University of Campinas (UNICAMP), Campinas São Paulo state, Brazil

**Keywords:** Hyaluronidase, Venom, Purification, *Vitalius dubius*

## Abstract

**Background:**

Venom hyaluronidase (Hyase) contributes to the diffusion of venom from the inoculation site. In this work, we purified and characterized Hyase from the venom of *Vitalius dubius* (Araneae, Theraphosidae), a large theraphosid found in southeastern Brazil. Venom obtained by electrical stimulation of adult male and female *V. dubius* was initially fractionated by gel filtration on a Superdex® 75 column. Active fractions were pooled and applied to a heparin-sepharose affinity column. The proteins were eluted with a linear NaCl gradient.

**Results:**

Active fractions were pooled and assessed for purity by SDS-PAGE and RP-HPLC. The physicochemical tests included optimum pH, heat stability, presence of isoforms, neutralization by flavonoids and assessment of commercial antivenoms. Hyase was purified and presented a specific activity of 148 turbidity-reducing units (TRU)/mg (venom: 36 TRU/mg; purification factor of ~4). Hyase displayed a molecular mass of 43 kDa by SDS-PAGE. Zymography in hyaluronic-acid-containing gels indicated an absence of enzyme isoforms. The optimum pH was 4-5, with highest activity at 37°C. Hyase was stable up to 60°C; but its activity was lost at higher temperatures and maintained after several freeze-thaw cycles. The NaCl concentration (up to 1 M) did not influence activity. Hyase had greater action towards hyaluronic acid compared to chondroitin sulfate, and was completely neutralized by polyvalent antiarachnid sera, but not by caterpillar, scorpion or snakes antivenoms.

**Conclusion:**

The neutralization by arachnid but not scorpion antivenom indicates that this enzyme shares antigenic epitopes with similar enzymes in other spider venoms. The biochemical properties of this Hyase are comparable to others described.

## Background

Spider venoms comprise complex mixtures of components of low molecular weight, peptides and proteins [1]. Among the enzymatic activities, collagenase and hyaluronidase (Hyase) are often found and were formerly attributed to matrix degradation [1].

Hyase activity is found in several spider species, such as *Cupiennius salei*, *Lycosa godeffroy*, *Lampona cylindrata/murina*, *Loxosceles recluse*, *Loxosceles rufescens*, *Loxosceles deserta*, *Loxosceles gaucho*, *Loxosceles laeta*, *Loxosceles recluse*, and *Loxosceles intermedia*[1-6]. The first report of hyaluronidasic activity was in venoms of the Brazilian spiders *Lycosa raptorial* and *Phoneutria nigriventer*[7,8]. The first Hyase to be purified from a Theraphosidae spider was the one from the tarantula *Dugesiella hentzi* (Girard), which was characterized and identified as the major venom component [9].

The clinical relevance of Hyases is not confined to a toxin-spreading factor, since it acts as an allergen in a manner similar to hymenoptera venoms. Hyase, phospholipase A2 and melitin were identified as the tree major causes of allergic reactions involved in bee stings, and phospholipase A1 and antigen 5 are the major allergens in wasp venoms [10,11].

Although tarantula bites to humans are relatively rare [12-14], these spider venoms represent a rich source of bioactive molecules for scientific interest, basic research and possible therapeutic applications [15-18].

The tarantula *Vitalius dubius* (Mello-Leitão, 1923) is characterized as nonaggressive and is found in the very populous area of southeastern Brazil [19]. Recent studies have shown that *V. dubius* venom has a complex biochemical and pharmacological composition, with different biological activities [20,21]. In the present work we describe the purification and characterization of the hyaluronidase present in its venom.

## Methods

### Reagents

Refined chemicals were purchased from Sigma Chemical Co. (USA) whereas heparin was purchased from Laboratório Cristália (Brazil). Molecular mass markers for SDS-PAGE were acquired from BioRad (USA). The resins for column chromatography were purchased from GE Healthcare Life Sciences (Sweden). The HPLC column was a Jupiter C18 (4.6 mm **×** 250 mm **×** 12 μm) acquired from Phenomenex (USA).

### Spiders and venom extraction

Specimens of *V. dubius* and *Phoneutria nigriventer* were provided by the Centers for Zoonosis Control for the cities in the region of Campinas, São Paulo state. The spiders were identified and kept in captivity, where they were fed cockroaches and water *ad libitum*. The tarantula venom was extracted as described by Rocha-e-Silva *et al.*[20] then lyophilized and stored at-80°C until use.

### Antisera

We used six antivenom sera produced by the Butantan Institute in São Paulo: (1) antiarachnid (*Phoneutria nigriventer*, *Loxosceles gaucho* and *Tityus serrulatus*); (2) antiscorpionic (*T. serrulatus*); (3) antilonomic (*Lonomia obliqua*); (4) antibothropic (*Bothrops alternatus*, *B. jararaca, B. jararacussu, B. moojeni* and *B. neuwiedi*); (5) anticrotalic (*Crotalus durissus terrificus* and *Crotalus durissus collilineatus*); and (6) antielapidic (*Micrurus frontalis* and *M. corallinus*).

### Enzymatic activity

Hyaluronidase activity was determined by a turbidimetric method [22]. The working solution for this trial consisted of a 200 μL buffer (0.2 M sodium acetate, pH 6.0, containing 0.15 M NaCl), 200 μL of substrate (hyaluronic acid from human umbilical cord 1 mg/mL in acetate buffer) and 100 μL of enzyme (20 μg) to give a total reaction volume of 500 μL. This mixture was incubated for 15 minutes at 37°C after which the reaction was stopped by adding 2 mL of hexadecyltrimethylammonium bromide in 2.5% NaOH. The resulting turbidity was read at 400 nm in a SpectraMax® 340 microplate reader (Molecular Devices, USA) after 30 minutes of incubation at room temperature. One unit of activity corresponded to the amount of enzyme that produced a 50% reduction in turbidity caused by 200 μg of substrate under the conditions described above.

### Purification of Hyaluronidase

The venom (10 mg) of *V. dubius* was dissolved in 0.1 M sodium acetate buffer, pH 6.0, containing 0.15 M NaCl, and fractionated by a Superdex® 75 gel filtration column (10 mm × 30 cm) balanced and eluted with the same buffer. Fractions of 1.5 mL each were collected at a flow rate of 1 mL per minute using an ÄKTA-Purifier chromatographic system (Pharmacia, Sweden). The elution profile was determined by monitoring the absorbance at 280 nm.

The hyaluronidase-containing fractions eluted from the previous step were applied to a heparin-sepharose column equilibrated with 0.1 M sodium acetate buffer, pH 6.0. The fractions were washed and eluted under a gradient of NaCl (0 to 1.0 M). The elution profile was determined by monitoring the absorbance at 280 nm whereas fractions (1.5 mL) containing the enzyme were stored at 8°C.

Hyase purity was evaluated by RP-HPLC chromatography using a resource RPC column (4.6 mm × 250 mm × 12 μm). The column was balanced with 0.1% trifluoracetic acid (TFA) and the enzyme was eluted with a linear gradient (0-100%) of 66% acetonitrile in 0.1% TFA. The elution profile was monitored at 280 nm.

### SDS-polyacrylamide gel electrophoresis (SDS-PAGE) and zymography

Electrophoresis was performed using 12% acrylamide or 5-20% gradients gels [23]. The existence of isoforms of Hyase was investigated as described by Cevallos *et al.*[24]. The hyaluronic acid was mixed into the SDS-polyacrylamide gels to obtain a final concentration of 0.6 mg/mL of non-polymerized solution.

During zymography, the electrophoretic separation phase was performed under the same conditions previously described (gels, buffers etc.), except for the samples, that were not warmed, to conserve their enzymatic activities. After Stains all staining (Sigma-Aldrich Co.), the gel was washed with a buffer containing 0.015 M Tris–HCl, 5% formamide and 20% isopropyl alcohol, pH 7.9, and photo documented.

### Physicochemical characterization of the enzyme

The optimum pH was determined by changing the buffers of the enzymatic turbidimetric assay as follows: 0.1 M sodium citrate, pH 3.0 to 6.0, 0.1 M sodium acetate, pH 4.0 to 6.0 and 0.1 M Tris–HCl, pH 5.5 to 8.0 (0.15 M NaCl was added to all buffers).

The optimal working temperature of the enzyme was evaluated by adjusting assay temperature between 10 and 70°C. The thermal stability of the enzyme was tested by pre-incubating enzyme solution for 15 minutes under a temperature ranging from 25 to 80°C.

To evaluate freezing longevity of the enzyme, separated aliquots were stored at-20°C and enzyme activity measured at the times 1, 2, 3, 6, 24, 72 hours and 7 and 15 days. Each aliquot was thawed once before the assay. To evaluate freezing cycles stability, a stock solution was stored, then thawed and refrozen for each enzyme assay, following same intervals in the freezing longevity test.

We investigated substrate specificity of the enzyme towards hyaluronic acid and chondroitin sulfate A. For this assay the Hyase activity was measured by quantifying the released sugars according to the colorimetric method of Reissig *et al.*[25].

The maximum reaction velocity (V_max_) and the substrate concentration that results in the half of the maximum velocity (K_m_) were assessed with fixed amounts of enzyme and variable substrate concentrations. The N-acetylamine release was quantified by the method of Reissig *et al*. [25]. The parameters were calculated using Lineweaver-Burk graphs, as previously described by Segel [26].

### Immunological comparisons

An ELISA test was used to assess cross-reactivity between the enzyme and the commercial sera against several venomous species of spiders, scorpions and snakes.

The ability of Instituto Butantan’s commercial antivenom to neutralize the enzymatic activity of *V. dubius* was assessed using the turbidimetric assay. The enzyme was pre-incubated with different volumes of each antivenom for 30 minutes at 37°C before measuring the enzyme activity. Control assays were performed using venom only.

### Statistics

The results were expressed as the mean ± SEM for the number of assays. Statistical comparisons were made using ANOVA followed by the Tukey test, with p < 0.05 indicating significance.

## Results

**Figure 1:**
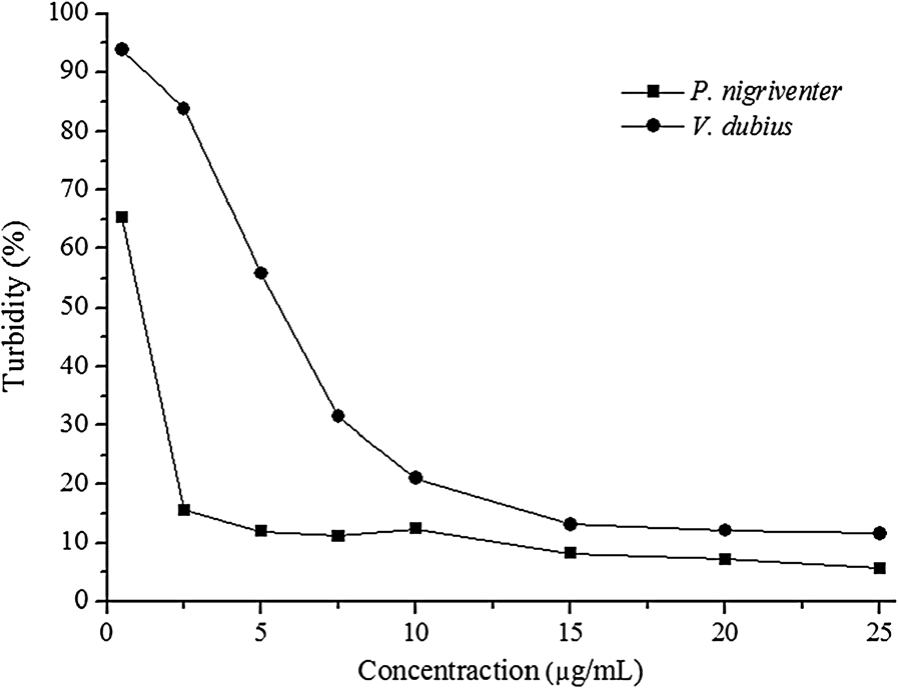
**Comparison of hyaluronidase activity present in the venom of *V. dubius *and *P. nigriventer*.** The values represent the mean ± S.E. (n = 6).

The Hyase activity of *V. dubius* venom was lower than that of *P. nigriventer* (with respective turbidities of 11.6 and 5.6%). The venom of *P. nigriventer* reached assay saturation at a concentration of 5 μg/mL, while *V. dubius* venom showed maximum activity at 15 μg/mL (Figure 1).

**Figure 2:**
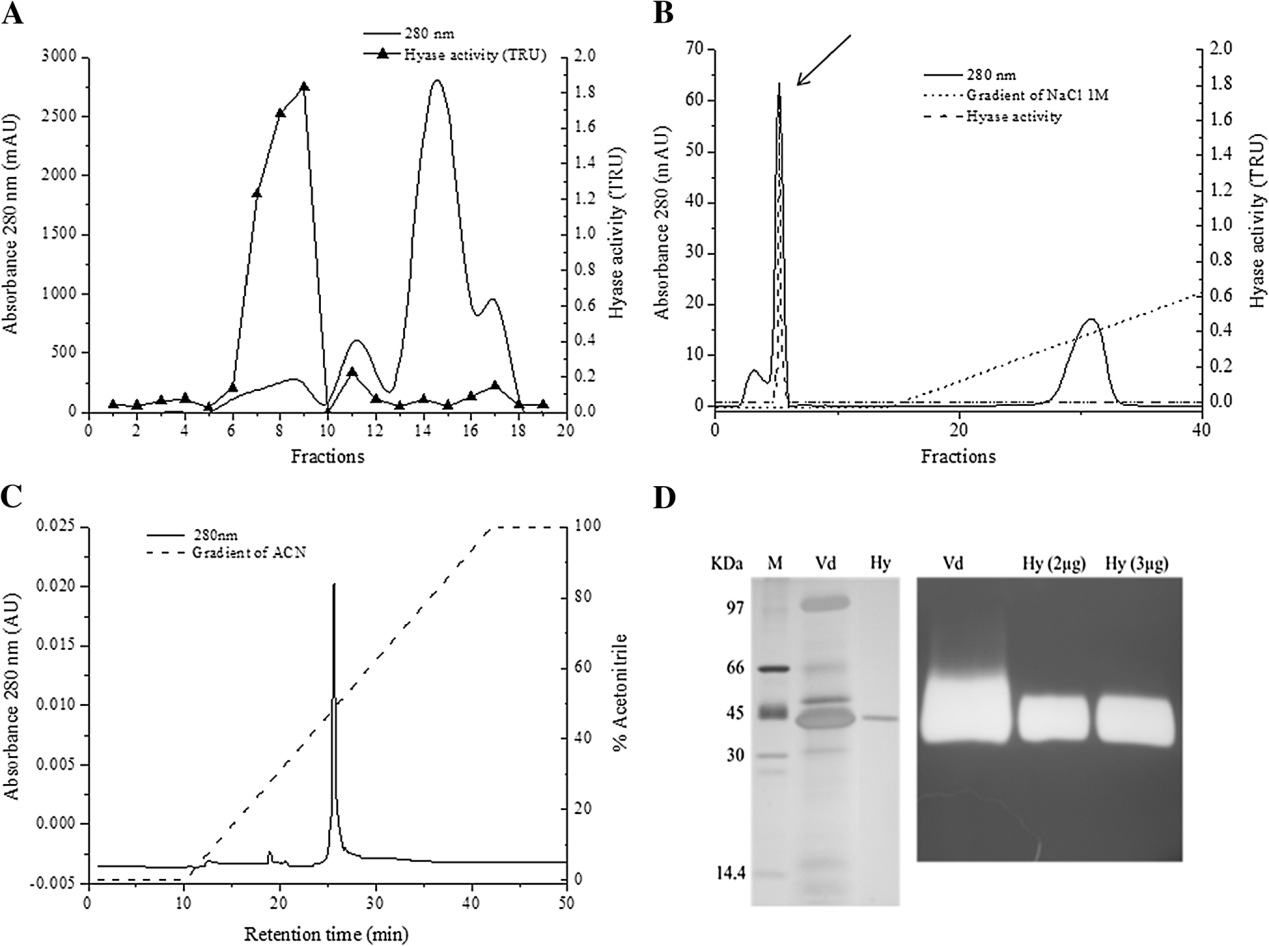
**Hyaluronidase purification from the venom of *V. dubius*. (A)** Gel filtration chromatography on Superdex® G-75 showing three active fractions. **(B)** Positive fractions from the previous step were applied to an affinity column of heparin-Sepharose (5 mL). The enzyme was detected at peak 2 (arrow), not retained by column. **(C)** C-18 reversed phase chromatography for final purification, using ACN gradient. **(D)** Left: SDS-PAGE of crude venom (Vd) and purified Hyase (Hy), using polyacrylamide gel 12% stained with silver nitrate. The estimated mass for the enzyme was 43 kDa. Right: crude venom and purified enzyme zymography. M = molecular weight markers.

Hyase purification was initiated through a size exclusion chromatography, which provided three active fractions (fractions 7, 8 and 9), pooled for the second stage (Figure 2A). In the following affinity chromatography, Hyase activity was detected in one fraction (Figure 2B) (fraction 5). Reversed-phase HPLC provided the purified Hyase (Figure 2C). The enzyme purity was confirmed by a 12% SDS-PAGE and zymography, which showed only one band around 43 kDa (Figure 2D).

About 850 μg of purified Hyase was obtained from 18 mg of crude venom protein, with an activity corresponding to 19.5% of the venom. The total activity significantly dwindled in the second stage (from 615.3 TRU to 124.4), but the specific activity augmented gradually during the purification process (148 U/mg). The purification factor had also increased (4.1).

**Figure 3:**
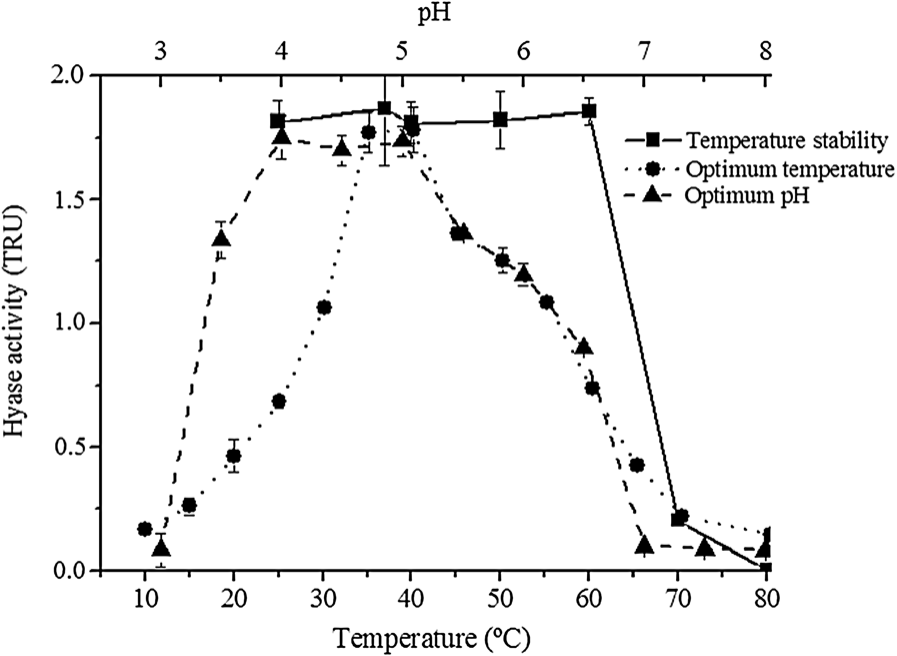
**Properties of hyaluronidase from *V. dubius*.** The purified enzyme showed maximum activity at pH between 4 and 5 and temperature from 35 and 40°C. Lower activity was observed starting from 25°C, decreasing gradually from 45°C onwards with total loss above 60°C. The points represent the mean ± S.E. (n = 6).

The investigation of the physicochemical characteristics of the purified enzyme showed maximum activity at pH between 4 and 5 and temperature from 35 and 40°C. Lower activity was observed at 25°C, decreasing gradually from 45°C onwards with total loss above 60°C (Figure 3).

**Figure 4:**
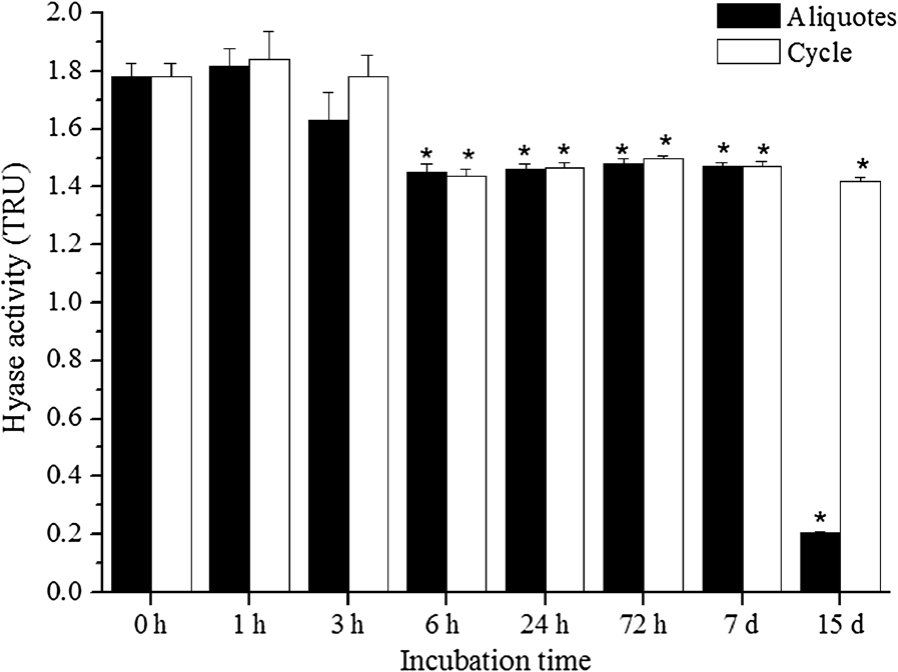
**Stability of purified Hyase after freeze-thaw cycles (-20°C): the activity remained at maximum after the first three freeze-thaw cycles (0, 1 and 3 hours), with a slight decrease after six hours, unchanged until 15 days.** Aliquots maintained their activity until the seventh day, losing activity after 15 days of freezing. The points represent mean ± SEM (n = 6), * p < 0.05.

Enzyme samples submitted to consecutive freeze-thaw cycles remained at maximum activity after the first three cycles (0, 1 and 3 hours), with a slight decrease after six hours, remaining stable until 15 days. Otherwise, individual frozen aliquots maintained their activity until the seventh day, losing activity after 15 days of freezing (Figure 4).

**Figure 5:**
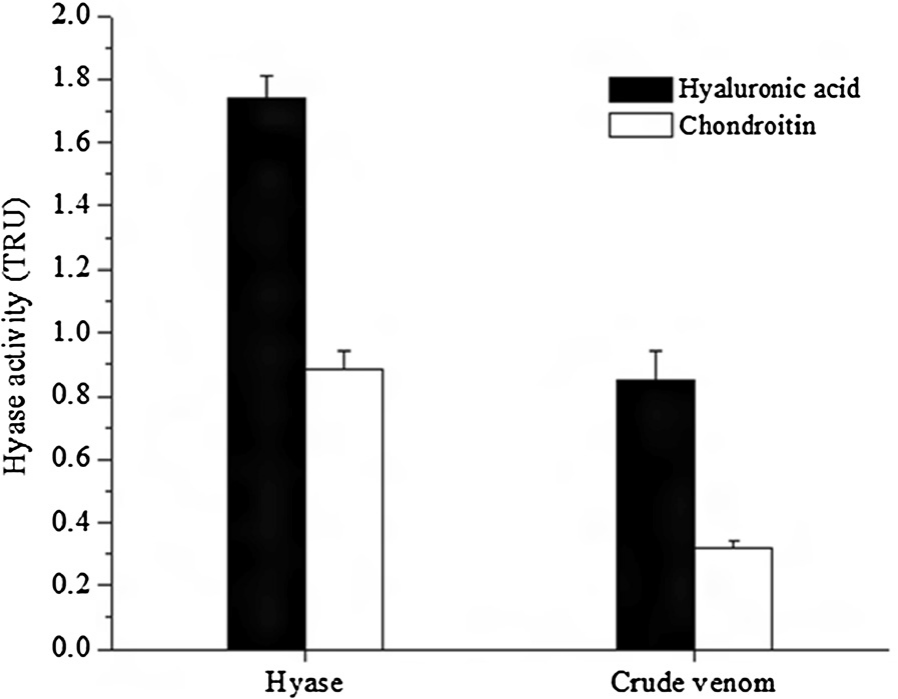
**Enzymatic activity of *V. dubius *venom (10 μg) and purified Hyase (5 μg) on hyaluronic acid and chondroitin sulfate A.** The points represent mean ± SEM (n = 6).

**Figure 6:**
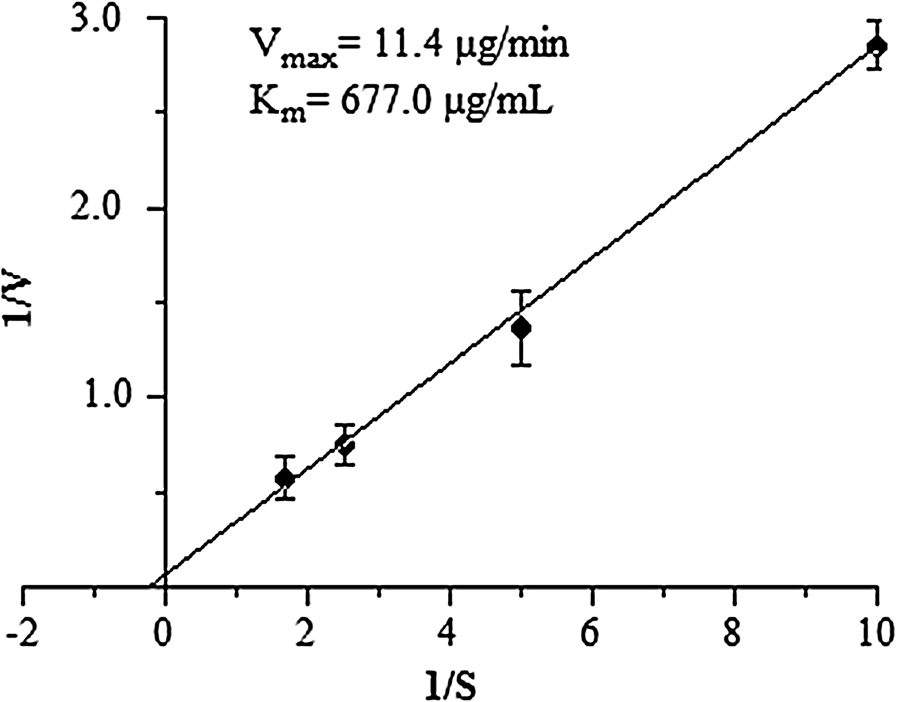
**Lineweaver-Burk graphs (1/S vs. 1/V) to Hyase V**_**max **_**and K**_**m **_**determination using hyaluronic acid as substrate.** The points represent mean ± SEM (n = 6).

The substrate specificity was evaluated in both purified enzyme and crude venom; it was observed that the Hyase has a higher specificity to hyaluronic acid, but a lower activity in the presence of chondroitin (Figure 5). The values obtained for V_max_ and K_m_ from *V. dubius-*purified Hyase were 11.4 μg/min and 677.0 μg/mL, respectively, as shown in Figure 6.

**Figure 7:**
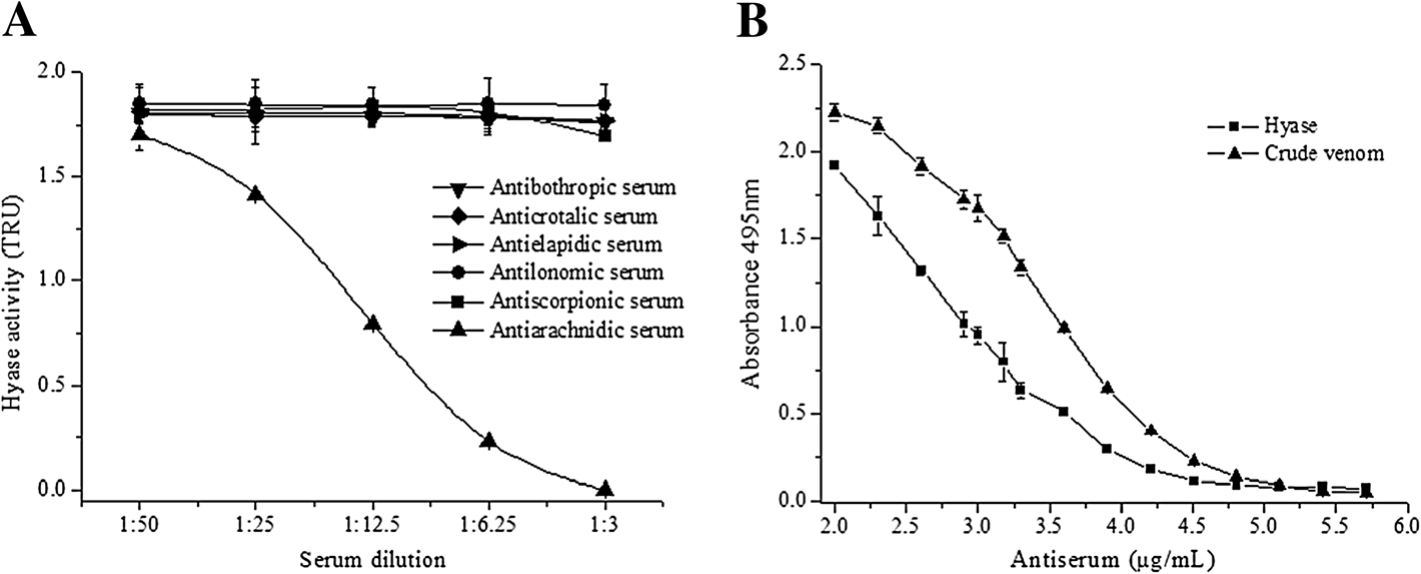
**Immunological comparisons. (A)** Dose-dependent neutralization of Hyase by anti-arachnidic serum. Antiscorpionic, antilonomic, antibothropic, anticrotalic and antielapidic sera were inefficient at neutralizing the enzymatic activity. **(B)** Reactivity of *V. dubius* venom (10 μg) and purified Hyase (5 μg) compared to anti-arachnidic serum on ELISA. The points represent mean ± SEM (n = 6).

Antiophidic, antiscorpionic and antilonomic serum were inefficient at neutralizing the enzymatic activity (Figure 7A). The antiarachnid serum against enzyme and crude venom neutralized Hyase activity in a dose-dependent manner (Figure 7B).

## Discussion

The hyaluronidases (Hyases) that have been isolated from various sources and extensively characterized comprise a group of enzymes described as 4-hyaluronate glucanohydrolase (EC 3.2.1.35, hyaluronoglucosamidase), hyaluronate-3-glucanohydrolase (EC 3.2.1.36, hyaluronoglucuronidase) and hyaluronatelyase (EC 4.2.2.1) [27]. The presence of this enzyme has been described in several human tissues, animal venoms, pathogenic organisms and cancers. Animal venom Hyases are structurally similar to those from the acrosome membrane of sperm, which play a fundamental role in mammalian fertilization, and to Hyases Hyal-1 and Hyal-2 of mammalian lysosomes [28-30]. Thus, venom Hyases are attributed the scattering function by facilitating the spread of toxins through glucosamine glycan hydrolysis in connective tissue, thereby yielding systemic poisoning [31].

As expected, the venom of *P. nigriventer* presents higher Hyase activity than that of *V. dubius*, due to the former’s abundant high-molecular-weight molecules, thus requiring a higher permeability to promote the dissemination of such components [1]. Moreover, the venom of *V. dubius* has few components of high molecular weight, but rather a predominance of low-weight molecules, such as peptides, thus less need of Hyase [20].

That molecular profile of *V. dubius* Hyase venom contributed to the enzyme purification process. In three chromatographic steps, we obtained the pure enzyme, while in the venoms from other animals–namely *Tityus serrulatus*, *Buthus martensi*, *Apis mellifera*, *Vespula vulgaris*, *Synanceja horrida* and *Lonomia obliqua*–more chromatographic steps were necessary for Hyase purification [11,27,28,32-34].

The enzyme yield (4.8%) was considered low after purification, mainly because the enzyme is present at small concentrations in the total venom. Similar yields (4.9%) can be obtained during the Hyase purification of the scorpion venom of *Tityus serrulatus*[27]. Otherwise, the purification from the venom of the scorpion *Palamneus gravimanus*, provided a higher yield, about 40% [31].

The optimum working temperature of purified *V. dubius* Hyase is around 37°C, which matches the physiological range of some of its largest prey, such as small rodents [35]. The enzyme loses its activity at 60°C, perhaps due to losses in the molecular structure, thus compromising its activity. Same hyaluronidase temperature range can be found in the venom activity presented by *Agkistrodon contortrix* and *Tityus serrulatus*[27,36].

The optimum pH found in *V. dubius* Hyase was between 4 and 5, the same described for *Buthus martensi* and *Palamneus gravimanus* enzymes [31,32]. In the Hyase of *Hippasa partita*, the best pH was observed to be 6 [37]. The finding of a pH between 4 and 5 can be explained by the preferential degradation of an acid substrate by the enzyme.

The *V. dubius* Hyase has a K_m_ of 677.0 μg/mL, indicating a comparatively low affinity of the substrate for the enzyme catalytic site, in contrast to the Hyases of *T. serrulatus* (69.7 μg/mL) and *P. gravimanus* (47.61 μg/mL) scorpions, but similar to the stonefish *Synanceja horrida* (709 μg/mL) [27,31,33].

Testing the specificity of substrates, we observed that *V. dubius* Hyase exerts greater activity on hyaluronic acid compared to chondroitin, although still positive for the latter. The chondroitin test was positive for bovine hyaluronidase but negative for Hyases purified from the venoms of *Hippasa partita* and *Agkistrodon contortrix*[36,37].

The neutralization of Hyase activity by antivenoms has not been widely studied. The experiments conducted in our laboratory showed that the venom Hyase of *V. dubius* was recognized by antiarachnid serum, in a dose-dependent manner. The lack of influence of the antiscorpionic sera, obtained from *Tytius* venoms that are used for antiarachnid production, attest that the above neutralization is specifically due to antibodies raised against *Loxosceles* and *Phoneutria* sp., pooled for antivenom preparation against the latter. Neutralization also failed with antilonomic and antisnake sera, reinforcing the reaction specificity of *V. dubius* Hyase against spider antivenoms. This indicates that immunological identities may differ between the Hyase of arachnids and those of snakes and caterpillars. This immunological relationship has also been reported for several other enzymes and toxins of venoms [38].

## Conclusions

Based on these findings, we conclude that the venom of *V. dubius* contains a Hyase with physicochemical and biochemical characteristics similar to other Hyases of venoms, although less potent. Nevertheless, the studied Hyase shares immunological features specifically with other spiders’ enzymes, rather than those of caterpillars and snakes.

## Ethics committee approval

The present study was approved by the Ethical Committee on Animal Research (UNICAMP) under the registration number 2167-1.

## Competing interests

The authors declare that there are no competing interests.

## Authors’ contributions

This work was developed by RS, with the assistance of MLT in the experiments and was mentored by TAARS and SH. All authors read and approved the final manuscript.
